# 2,4-Dichloro-6-(8-quinolylamino­methyl­ene)cyclo­hexa-2,4-dien-1-one methanol solvate

**DOI:** 10.1107/S1600536810002345

**Published:** 2010-01-23

**Authors:** Takashi Shibahara, Masayuki Takahashi, Atsushi Maekawa, Hideaki Takagi

**Affiliations:** a Department of Chemistry, Okayama University of Science, Ridai-cho, Kita-ku, Okayama 700-0005, Japan; b Department of International Conservation Studies for Cultural Properties, Kibi International University, Igamachi 8, Takahashi-shi, Okayama 716-8508, Japan

## Abstract

The main mol­ecule of the title methanol solvate, C_16_H_10_Cl_2_N_2_O·CH_3_OH, exists in the keto form and the C=O and N—H bonds are mutually *cis* in the crystal structure. The dihedral angle between the quinoline and benzene rings is 11.17 (3)°. A bifurcated intra­molecular N—H⋯(O,N) hydrogen bond is present as well as an O—H⋯O hydrogen bond. In the crystal, C—H⋯O inter­actions link the 3,5-dichloro­salicyl­idene-8-amino­quinoline and methanol mol­ecules.

## Related literature

For a related structure, see: Sakane *et al.* (2006 ▶).
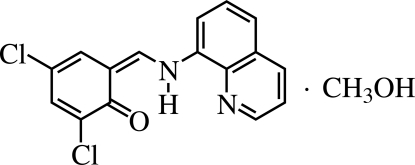


## Experimental

### 

#### Crystal data

C_16_H_10_Cl_2_N_2_O·CH_4_O
*M* *_r_* = 349.22
Triclinic, 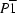

*a* = 7.044 (2) Å
*b* = 8.139 (3) Å
*c* = 13.935 (5) Å
α = 88.030 (11)°
β = 80.205 (9)°
γ = 73.611 (7)°
*V* = 755.2 (4) Å^3^
*Z* = 2
Mo *K*α radiation
μ = 0.44 mm^−1^
*T* = 93 K
0.71 × 0.24 × 0.18 mm

#### Data collection

Rigaku Mercury diffractometer
Absorption correction: multi-scan (*REQAB*; Jacobson, 1998 ▶) *T*_min_ = 0.745, *T*_max_ = 0.925
7177 measured reflections
3357 independent reflections
3262 reflections with *F* ^2^ > 2σ(*F* ^2^)
*R*_int_ = 0.022

#### Refinement

*R*[*F* ^2^ > 2σ(*F* ^2^)] = 0.028
*wR*(*F* ^2^) = 0.078
*S* = 1.01
3357 reflections
209 parameters
H-atom parameters not refined
Δρ_max_ = 0.42 e Å^−3^
Δρ_min_ = −0.28 e Å^−3^

### 

Data collection: *CrystalClear* (Rigaku, 1999 ▶); cell refinement: *CrystalClear*; data reduction: *CrystalStructure* (Rigaku/MSC, 2007 ▶); program(s) used to solve structure: *SIR92* (Altomare *et al.*, 1994 ▶); program(s) used to refine structure: *SHELXL97* (Sheldrick, 2008 ▶); molecular graphics: *CrystalStructure*; software used to prepare material for publication: *CrystalStructure*.

## Supplementary Material

Crystal structure: contains datablocks global, I. DOI: 10.1107/S1600536810002345/pv2250sup1.cif

Structure factors: contains datablocks I. DOI: 10.1107/S1600536810002345/pv2250Isup2.hkl

Additional supplementary materials:  crystallographic information; 3D view; checkCIF report

## Figures and Tables

**Table 1:** Hydrogen-bond geometry (Å, °)

*D*—H⋯*A*	*D*—H	H⋯*A*	*D*⋯*A*	*D*—H⋯*A*
O2—H14⋯O1	0.82	1.95	2.7694 (14)	179
N2—H7⋯O1	0.86	1.92	2.6094 (12)	137
N2—H7⋯N1	0.86	2.28	2.6758 (13)	108
C8—H6⋯O2^i^	0.97	2.70	3.6425 (14)	166
C10—H8⋯O2^i^	0.98	2.27	3.2026 (13)	160

Symmetry code: (i) 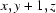.

**Fig. 1.**
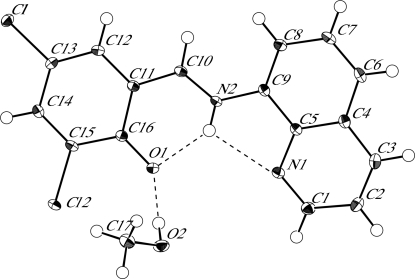
Molecular configuration and atom-numbering scheme for (I).CH3OH with displacement ellipsoids drawn at the 50% probability level. Hydrogen bonds are shown as dashed lines.

**Fig. 2.**
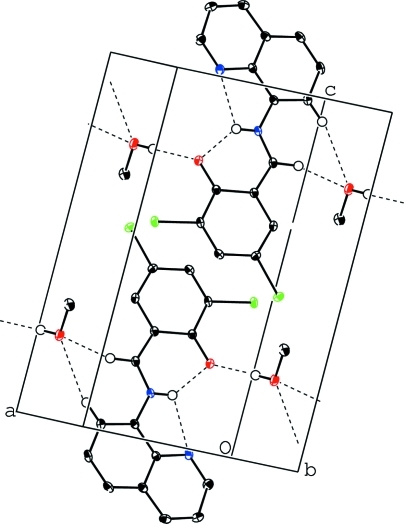
Molecular packing of (I).CH3OH in the unit cell.

## supplementary crystallographic information

### Comment

In the present work, the title compound, (I), was prepared and its crystal structure determined, to explore the modification effect of Schiff base ligand on the fluorescence of metal complexes of 2-hydroxy-1-naphthaldehydene-8-aminoquinoline (C_20_H_14_N_2_O), (II) (Sakane *et al.*, 2006). The molecule of (I) (Fig. 1) exists in the keto form and the C═O and N—H bonds are mutually *cis* which is similar to that found in the structure of (II). In the structure of (I), *N*—*H*···O carbonyl and *N*—*H*···N pyridine intramolecular hydrogen bonds exist (Table 1). In addition, there is a formal intermolecular hydrogen-bonding association between the molecules of 3,5-dichlorosalicylidene-8-aminoquinoline and  methanol solvate (Table 1 and Fig. 2).

### Experimental

Refluxing a suspension of 8-aminoquinoline (145 mg, 1.0 mmol) and 3,5-dichloro-salicylaldehyde (191 mg, 1.0 mmol) in methanol (3 ml) at 338 K for one hour gave vivid red powder. Slow evaporation of the supernatant solution gave vivid red plate like crystals of C_16_H_10_Cl_2_N_2_O (I).CH_3_OH. Yield 302 mg (95%). Anal. Found: C, 58.03; H, 3.73; N, 8.21%. Calcd for C_17_H_14_N_2_O_2_: C, 58.47; H, 4.04; N, 8.02%.

### Refinement

The positions of all H atoms were located from difference maps and refined with restrained distances (N–H = 0.86 Å; C–H = 0.92-1.00 Å). The isotropic displacement parameters for H atoms were fixed at 1.2U_eq_ of their carrier atoms.

### Crystal data

**Table:**

C_16_H_10_Cl_2_N_2_O·CH_4_O	*Z* = 2
*M**_r_* = 349.22	*F*(000) = 360.00
Triclinic, *P*1	*D*_x_ = 1.536 Mg m^−^^3^
Hall symbol: -P 1	Mo *K*α radiation, λ = 0.71070 Å
*a* = 7.044 (2) Å	Cell parameters from 1999 reflections
*b* = 8.139 (3) Å	θ = 5.6–27.5°
*c* = 13.935 (5) Å	µ = 0.44 mm^−^^1^
α = 88.030 (11)°	*T* = 93 K
β = 80.205 (9)°	Platelet, red
γ = 73.611 (7)°	0.71 × 0.24 × 0.18 mm
*V* = 755.2 (4) Å^3^	

### Data collection

**Table:**

Rigaku Mercury diffractometer	3357 independent reflections
Radiation source: Mo Kα	3262 reflections with *F*^2^ > 2σ(*F*^2^)
Detector resolution: 14.63 pixels mm^-1^	*R*_int_ = 0.022
ω scans	θ_max_ = 27.5°
Absorption correction: multi-scan (REQAB; Jacobson, 1998)	*h* = −8→9
*T*_min_ = 0.745, *T*_max_ = 0.925	*k* = −10→10
7177 measured reflections	*l* = −18→17

### Refinement

**Table:**

Refinement on *F*^2^	H-atom parameters not refined
*R*[*F*^2^ > 2σ(*F*^2^)] = 0.028	*w* = 1/[σ^2^(*F*_o_^2^) + (0.043*P*)^2^ + 0.4083*P*] where *P* = (*F*_o_^2^ + 2*F*_c_^2^)/3
*wR*(*F*^2^) = 0.078	(Δ/σ)_max_ = 0.001
*S* = 1.01	Δρ_max_ = 0.42 e Å^−^^3^
3357 reflections	Δρ_min_ = −0.28 e Å^−^^3^
209 parameters	

### Special details

**Table:**

Geometry. The dihedral angle between the quinoline (C1~C9, N1) and the benzene rings (C11~C16) is 11.17 (3)°: Mean deviations of the atoms from the former and latter planes are 0.014 and 0.004 Å, respectively.
Refinement. Refinement was performed using all reflections. The weighted *R*-factor (*wR*) and goodness of fit (*S*) are based on *F*^2^. *R*-factor (gt) are based on *F*. The threshold expression of *F*^2^ > 2.0 σ(*F*^2^) is used only for calculating *R*-factor (gt).

### Fractional atomic coordinates and isotropic or equivalent isotropic displacement parameters (Å^2^)

**Table:**

	*x*	*y*	*z*	*U*_iso_*/*U*_eq_	
Cl(2)	1.24813 (4)	0.00187 (3)	0.581434 (19)	0.01543 (8)	
Cl(1)	1.28180 (4)	0.62464 (3)	0.452063 (19)	0.01392 (8)	
O(1)	1.03744 (12)	0.19010 (10)	0.76600 (6)	0.01351 (16)	
O(2)	0.81786 (14)	−0.04501 (11)	0.77301 (6)	0.01778 (18)	
N(1)	0.83476 (14)	0.24199 (12)	1.01752 (7)	0.01204 (18)	
N(2)	0.88903 (14)	0.46400 (12)	0.87656 (7)	0.01041 (17)	
C(1)	0.80423 (17)	0.13097 (15)	1.08523 (9)	0.0146 (2)	
C(2)	0.68177 (18)	0.17741 (16)	1.17744 (8)	0.0161 (2)	
C(3)	0.59125 (17)	0.34651 (16)	1.19956 (8)	0.0149 (2)	
C(4)	0.62125 (16)	0.47193 (15)	1.12960 (8)	0.0119 (2)	
C(5)	0.74388 (16)	0.41182 (14)	1.03902 (8)	0.0105 (2)	
C(6)	0.53722 (17)	0.65040 (15)	1.14652 (8)	0.0146 (2)	
C(7)	0.57568 (17)	0.76438 (14)	1.07635 (9)	0.0150 (2)	
C(8)	0.69577 (17)	0.70710 (14)	0.98534 (8)	0.0128 (2)	
C(9)	0.77592 (16)	0.53372 (14)	0.96660 (8)	0.0104 (2)	
C(10)	0.94877 (16)	0.54862 (14)	0.80119 (8)	0.0111 (2)	
C(11)	1.05243 (16)	0.46773 (14)	0.71172 (8)	0.0109 (2)	
C(16)	1.09174 (16)	0.28530 (14)	0.69891 (8)	0.0106 (2)	
C(15)	1.19770 (17)	0.21966 (14)	0.60378 (8)	0.0118 (2)	
C(14)	1.25470 (16)	0.32146 (14)	0.53118 (8)	0.0118 (2)	
C(13)	1.20940 (16)	0.49959 (14)	0.54743 (8)	0.0114 (2)	
C(12)	1.11163 (16)	0.57250 (14)	0.63530 (8)	0.0114 (2)	
C(17)	0.72759 (19)	−0.02464 (16)	0.68811 (9)	0.0184 (2)	
H(1)	0.8725	0.0177	1.0721	0.018*	
H(2)	0.6749	0.0946	1.2238	0.019*	
H(3)	0.5055	0.3897	1.2617	0.018*	
H(4)	0.4564	0.6860	1.2066	0.018*	
H(5)	0.5203	0.8855	1.0900	0.018*	
H(6)	0.7140	0.7905	0.9363	0.015*	
H(7)	0.9237	0.3548	0.8700	0.012*	
H(8)	0.9221	0.6724	0.8071	0.013*	
H(10)	1.3178	0.2802	0.4703	0.014*	
H(9)	1.0851	0.6895	0.6459	0.014*	
H(11)	0.6033	0.0671	0.6913	0.022*	
H(12)	0.6853	−0.1289	0.6783	0.022*	
H(13)	0.8203	−0.0129	0.6303	0.022*	
H(14)	0.8844	0.0234	0.7709	0.021*	

### Atomic displacement parameters (Å^2^)

**Table:**

	*U*^11^	*U*^22^	*U*^33^	*U*^12^	*U*^13^	*U*^23^
Cl(2)	0.02066 (16)	0.00883 (14)	0.01551 (14)	−0.00396 (10)	0.00090 (10)	−0.00332 (10)
Cl(1)	0.01419 (14)	0.01408 (14)	0.01329 (14)	−0.00503 (10)	−0.00052 (10)	0.00295 (10)
O(1)	0.0180 (4)	0.0099 (3)	0.0130 (3)	−0.0053 (3)	−0.0011 (3)	−0.0001 (2)
O(2)	0.0235 (4)	0.0142 (4)	0.0197 (4)	−0.0104 (3)	−0.0062 (3)	0.0020 (3)
N(1)	0.0115 (4)	0.0102 (4)	0.0138 (4)	−0.0022 (3)	−0.0018 (3)	−0.0008 (3)
N(2)	0.0114 (4)	0.0082 (4)	0.0114 (4)	−0.0023 (3)	−0.0015 (3)	−0.0019 (3)
C(1)	0.0127 (5)	0.0123 (5)	0.0171 (5)	−0.0012 (4)	−0.0018 (4)	0.0009 (4)
C(2)	0.0153 (5)	0.0181 (5)	0.0141 (5)	−0.0044 (4)	−0.0017 (4)	0.0046 (4)
C(3)	0.0126 (5)	0.0200 (5)	0.0117 (5)	−0.0043 (4)	−0.0011 (4)	0.0001 (4)
C(4)	0.0102 (4)	0.0148 (5)	0.0114 (4)	−0.0038 (4)	−0.0026 (3)	−0.0019 (4)
C(5)	0.0089 (4)	0.0113 (5)	0.0117 (4)	−0.0027 (3)	−0.0029 (3)	−0.0015 (3)
C(6)	0.0131 (5)	0.0164 (5)	0.0136 (5)	−0.0031 (4)	−0.0004 (4)	−0.0057 (4)
C(7)	0.0148 (5)	0.0110 (5)	0.0187 (5)	−0.0025 (4)	−0.0018 (4)	−0.0054 (4)
C(8)	0.0131 (4)	0.0114 (5)	0.0145 (5)	−0.0041 (4)	−0.0026 (4)	−0.0012 (3)
C(9)	0.0087 (4)	0.0118 (5)	0.0111 (4)	−0.0030 (3)	−0.0019 (3)	−0.0021 (3)
C(10)	0.0108 (4)	0.0099 (4)	0.0135 (4)	−0.0031 (3)	−0.0034 (3)	−0.0010 (3)
C(11)	0.0107 (4)	0.0101 (4)	0.0123 (4)	−0.0030 (3)	−0.0028 (3)	−0.0006 (3)
C(16)	0.0104 (4)	0.0104 (4)	0.0116 (4)	−0.0032 (3)	−0.0029 (3)	−0.0004 (3)
C(15)	0.0121 (4)	0.0090 (4)	0.0144 (5)	−0.0028 (3)	−0.0024 (3)	−0.0027 (3)
C(14)	0.0107 (4)	0.0132 (5)	0.0115 (4)	−0.0031 (4)	−0.0015 (3)	−0.0024 (3)
C(13)	0.0109 (4)	0.0121 (4)	0.0119 (4)	−0.0043 (3)	−0.0026 (3)	0.0027 (3)
C(12)	0.0113 (4)	0.0088 (4)	0.0145 (5)	−0.0030 (3)	−0.0032 (3)	−0.0001 (3)
C(17)	0.0188 (5)	0.0170 (5)	0.0199 (5)	−0.0049 (4)	−0.0043 (4)	−0.0017 (4)

### Geometric parameters (Å, °)

**Table:**

Cl(2)—C(15)	1.7350 (11)	C(11)—C(12)	1.4220 (15)
Cl(1)—C(13)	1.7411 (11)	C(16)—C(15)	1.4446 (14)
O(1)—C(16)	1.2714 (14)	C(15)—C(14)	1.3650 (16)
O(2)—C(17)	1.4193 (16)	C(14)—C(13)	1.4114 (15)
N(1)—C(1)	1.3172 (15)	C(13)—C(12)	1.3631 (14)
N(1)—C(5)	1.3690 (13)	O(2)—H(14)	0.820
N(2)—C(9)	1.4088 (13)	N(2)—H(7)	0.856
N(2)—C(10)	1.3107 (14)	C(1)—H(1)	0.920
C(1)—C(2)	1.4179 (15)	C(2)—H(2)	0.923
C(2)—C(3)	1.3649 (16)	C(3)—H(3)	0.980
C(3)—C(4)	1.4214 (16)	C(6)—H(4)	0.933
C(4)—C(5)	1.4184 (14)	C(7)—H(5)	0.965
C(4)—C(6)	1.4166 (15)	C(8)—H(6)	0.965
C(5)—C(9)	1.4261 (15)	C(10)—H(8)	0.975
C(6)—C(7)	1.3709 (16)	C(14)—H(10)	0.915
C(7)—C(8)	1.4122 (15)	C(12)—H(9)	0.929
C(8)—C(9)	1.3798 (15)	C(17)—H(11)	0.973
C(10)—C(11)	1.4130 (14)	C(17)—H(12)	0.996
C(11)—C(16)	1.4431 (16)	C(17)—H(13)	0.967
			
C(1)—N(1)—C(5)	117.32 (9)	C(14)—C(13)—C(12)	121.14 (10)
C(9)—N(2)—C(10)	126.88 (9)	C(11)—C(12)—C(13)	119.52 (10)
N(1)—C(1)—C(2)	123.85 (10)	C(17)—O(2)—H(14)	108.2
C(1)—C(2)—C(3)	119.18 (11)	C(9)—N(2)—H(7)	117.1
C(2)—C(3)—C(4)	119.30 (9)	C(10)—N(2)—H(7)	116.0
C(3)—C(4)—C(5)	117.05 (9)	N(1)—C(1)—H(1)	117.0
C(3)—C(4)—C(6)	123.46 (9)	C(2)—C(1)—H(1)	119.1
C(5)—C(4)—C(6)	119.49 (10)	C(1)—C(2)—H(2)	119.7
N(1)—C(5)—C(4)	123.28 (10)	C(3)—C(2)—H(2)	120.8
N(1)—C(5)—C(9)	118.02 (9)	C(2)—C(3)—H(3)	124.5
C(4)—C(5)—C(9)	118.70 (9)	C(4)—C(3)—H(3)	116.2
C(4)—C(6)—C(7)	120.33 (9)	C(4)—C(6)—H(4)	117.5
C(6)—C(7)—C(8)	121.05 (9)	C(7)—C(6)—H(4)	122.2
C(7)—C(8)—C(9)	119.58 (10)	C(6)—C(7)—H(5)	119.1
N(2)—C(9)—C(5)	115.40 (9)	C(8)—C(7)—H(5)	119.9
N(2)—C(9)—C(8)	123.77 (10)	C(7)—C(8)—H(6)	118.9
C(5)—C(9)—C(8)	120.81 (9)	C(9)—C(8)—H(6)	121.4
N(2)—C(10)—C(11)	122.74 (10)	N(2)—C(10)—H(8)	118.8
C(10)—C(11)—C(16)	120.40 (10)	C(11)—C(10)—H(8)	118.4
C(10)—C(11)—C(12)	117.76 (10)	C(15)—C(14)—H(10)	122.9
C(16)—C(11)—C(12)	121.83 (9)	C(13)—C(14)—H(10)	117.2
O(1)—C(16)—C(11)	122.66 (9)	C(11)—C(12)—H(9)	119.8
O(1)—C(16)—C(15)	122.74 (10)	C(13)—C(12)—H(9)	120.6
C(11)—C(16)—C(15)	114.60 (9)	O(2)—C(17)—H(11)	115.5
Cl(2)—C(15)—C(16)	117.70 (8)	O(2)—C(17)—H(12)	108.9
Cl(2)—C(15)—C(14)	119.29 (8)	O(2)—C(17)—H(13)	112.0
C(16)—C(15)—C(14)	123.00 (10)	H(11)—C(17)—H(12)	103.0
C(15)—C(14)—C(13)	119.89 (9)	H(11)—C(17)—H(13)	109.7
Cl(1)—C(13)—C(14)	118.23 (7)	H(12)—C(17)—H(13)	107.0
Cl(1)—C(13)—C(12)	120.63 (8)		
			
C(1)—N(1)—C(5)—C(4)	0.26 (18)	C(6)—C(7)—C(8)—C(9)	−0.04 (15)
C(1)—N(1)—C(5)—C(9)	179.81 (11)	C(7)—C(8)—C(9)—N(2)	176.74 (11)
C(5)—N(1)—C(1)—C(2)	0.84 (19)	C(7)—C(8)—C(9)—C(5)	−1.86 (19)
C(9)—N(2)—C(10)—C(11)	−176.33 (11)	N(2)—C(10)—C(11)—C(16)	1.95 (18)
C(10)—N(2)—C(9)—C(5)	−175.47 (11)	N(2)—C(10)—C(11)—C(12)	−179.38 (11)
C(10)—N(2)—C(9)—C(8)	5.9 (2)	C(10)—C(11)—C(16)—O(1)	−0.39 (18)
N(1)—C(1)—C(2)—C(3)	−1.1 (2)	C(10)—C(11)—C(16)—C(15)	179.62 (10)
C(1)—C(2)—C(3)—C(4)	0.22 (19)	C(10)—C(11)—C(12)—C(13)	−179.06 (11)
C(2)—C(3)—C(4)—C(5)	0.77 (18)	C(16)—C(11)—C(12)—C(13)	−0.41 (17)
C(2)—C(3)—C(4)—C(6)	−178.65 (12)	C(12)—C(11)—C(16)—O(1)	−179.01 (11)
C(3)—C(4)—C(5)—N(1)	−1.05 (18)	C(12)—C(11)—C(16)—C(15)	1.00 (16)
C(3)—C(4)—C(5)—C(9)	179.40 (11)	O(1)—C(16)—C(15)—Cl(2)	0.90 (16)
C(3)—C(4)—C(6)—C(7)	178.72 (12)	O(1)—C(16)—C(15)—C(14)	179.36 (11)
C(5)—C(4)—C(6)—C(7)	−0.68 (19)	C(11)—C(16)—C(15)—Cl(2)	−179.10 (8)
C(6)—C(4)—C(5)—N(1)	178.39 (11)	C(11)—C(16)—C(15)—C(14)	−0.64 (17)
C(6)—C(4)—C(5)—C(9)	−1.16 (18)	Cl(2)—C(15)—C(14)—C(13)	178.14 (9)
N(1)—C(5)—C(9)—N(2)	4.16 (16)	C(16)—C(15)—C(14)—C(13)	−0.30 (18)
N(1)—C(5)—C(9)—C(8)	−177.13 (11)	C(15)—C(14)—C(13)—Cl(1)	−179.04 (9)
C(4)—C(5)—C(9)—N(2)	−176.26 (11)	C(15)—C(14)—C(13)—C(12)	0.97 (18)
C(4)—C(5)—C(9)—C(8)	2.45 (18)	Cl(1)—C(13)—C(12)—C(11)	179.40 (9)
C(4)—C(6)—C(7)—C(8)	1.31 (19)	C(14)—C(13)—C(12)—C(11)	−0.61 (17)

### Hydrogen-bond geometry (Å, °)

**Table:**

*D*—H···*A*	*D*—H	H···*A*	*D*···*A*	*D*—H···*A*
O2—H14···O1	0.820	1.950	2.7694 (14)	179
N2—H7···O1	0.856	1.916	2.6094 (12)	137
N2—H7···N1	0.856	2.284	2.6758 (13)	108
C8—H6···O2^i^	0.965	2.699	3.6425 (14)	166
C10—H8···O2^i^	0.975	2.268	3.2026 (13)	160

Symmetry codes: (i) *x*, *y*+1, *z*.
